# Application of Solid Lipid Nanoparticles to Improve the Efficiency of Anticancer Drugs

**DOI:** 10.3390/nano9030474

**Published:** 2019-03-22

**Authors:** Laura Bayón-Cordero, Itziar Alkorta, Lide Arana

**Affiliations:** 1 Biochemistry and Molecular Biology Department, University of the Basque Country (UPV/EHU), Barrio Sarriena S/N, 48940 Leioa, Spain; lbayon95@gmail.com (L.B.-C.); itzi.alkorta@ehu.eus (I.A.); 2 Instituto Biofisika (CSIC, UPV/EHU), Barrio Sarriena S/N, 48940 Leioa, Spain

**Keywords:** solid lipid nanoparticles, drug delivery, cancer, tumor, chemotherapy

## Abstract

Drug delivery systems have opened new avenues to improve the therapeutic effects of already-efficient molecules. Particularly, Solid Lipid Nanoparticles (SLNs) have emerged as promising nanocarriers in cancer therapy. SLNs offer remarkable advantages such as low toxicity, high bioavailability of drugs, versatility of incorporation of hydrophilic and lipophilic drugs, and feasibility of large-scale production. Their molecular structure is crucial to obtain high quality SLN preparations and it is determined by the relationship between the composition and preparation method. Additionally, SLNs allow overcoming several physiological barriers that hinder drug delivery to tumors and are also able to escape multidrug resistance mechanisms, characteristic of cancer cells. Focusing on cell delivery, SLNs can improve drug delivery to target cells by different mechanisms, such as passive mechanisms that take advantage of the tumor microenvironment, active mechanisms by surface modification of SLNs, and codelivery mechanisms. SLNs can incorporate many different drugs and have proven to be effective in different types of tumors (i.e., breast, lung, colon, liver, and brain), corroborating their potential. Finally, it has to be taken into account that there are still some challenges to face in the application of SLNs in anticancer treatments but their possibilities seem to be high.

## 1. Introduction

### 1.1. Drug Delivery Systems

The development in the last fifty years of biochemistry, molecular biology, biophysics, and cell biology, among other scientific disciplines, has led to significant advances in biomedicine based on the molecular knowledge of many diseases. This has made possible the development of therapeutic molecules effectively directed to the origin of the problem: the molecular and cellular processes that lead to the development of diseases. This is particularly important in cancer research where continuous progresses are made. Unfortunately, the development of new drugs is not enough to improve their effects on therapy. Some drugs are poorly soluble in water and cannot be administered unless they are encapsulated into drug carriers. In other occasions, drugs cannot permeate cell membranes and as a consequence the concentration at the target site is insufficient. To overcome this, high doses of drugs are required, causing high toxicity and many undesired side effects. Consequently, a targeted drug delivery system could selectively carry sufficient drug concentrations into the targeted tissue (or cell), improving its bioavailability and reducing the associated side effects due to high doses. In this regard, nanotechnology has expanded the therapeutic options of *a priori* efficient molecules by developing efficient Drug Delivery Systems (DDS). In fact, the application of nanotechnology in the administration and delivery of drugs has brought significant advances in medicine, promoting the emergence of a new field: nanomedicine [1]. Diverse DDS have been developed by combining their composition (organic, inorganic, or hybrid), size (small or large), shapes (sphere, rod, or cube), and surface properties (surface charge, functional groups, PEGylation or other coating, and attachment of targeting moieties) [2]. By doing so, different properties of therapeutic molecules, such as solubility, pharmacokinetic profile, cellular uptake, biodistribution pattern, circulation time, and clearance mechanisms, can be improved [1,2]. Thanks to the fast development of nanotechnology, there is a vast variety of nanocarriers that offer a wide spectrum of options to treat each therapeutic problem by a tailored approach. Among the most studied organic DDS it is worth mentioning liposomes, dendrimers, polymeric nanoparticles or micelles, polymer–drug/protein conjugates, and lipid nanoparticles. Additionally, some inorganic nanocarriers such as carbon nanotubes and mesoporous silica nanoparticles have been developed [1,2,3].

### 1.2. Solid Lipid Nanoparticles

**Figure 1:**
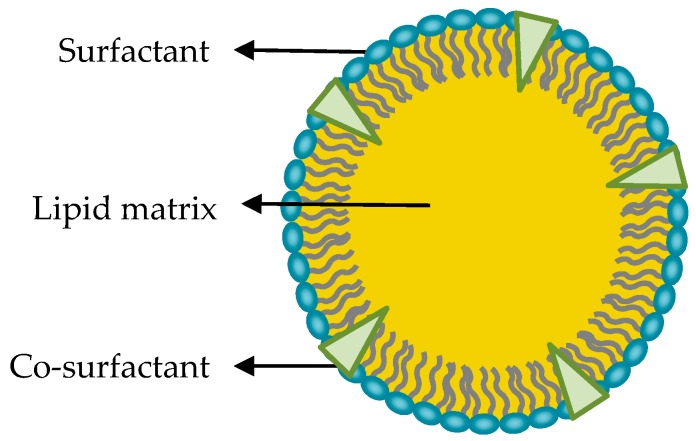
Proposed model of solid lipid nanoparticles structure. Schematic representation of solid lipid nanoparticle (SLN) structure, showing the surfactant, cosurfactant, and the solid lipid matrix.

It is well known that lipid-based nanoparticles are less toxic and biocompatible compared to inorganic or polymeric nanoparticles [4,5]. In particular, solid lipid nanoparticles (SLNs) have emerged as an effective and promising alternative. They are colloidal particles of submicron size, with a diameter between 50 and 1000 nm. They are made of a lipid matrix solid at physiological temperature, surfactants and, in some occasions, by cosurfactants (Figure 1) [6].

SLNs can be produced by different methods described exhaustively in the bibliography, such as high shear homogenization and ultrasound, high-pressure homogenization, hot homogenization, cold homogenization, solvent emulsification/evaporation, and microemulsion [6,7,8,9]. Among them, the microemulsion method stands out for being an easy method that does not need very sophisticated equipment or high-energy input and avoids the use of organic solvents. All these advantages make the production of SLNs at large scale technically and economically feasible [10]. Nonetheless, the correct composition is essential for the formation of microemulsions (thermodynamically stable and transparent mixtures), and therefore the optimization of the mixture is required. 

### 1.3. Composition and Structure of Solid Lipid Nanoparticles

SLNs are versatile nanocarriers that have been applied to improve the therapeutic effect of different molecules. However, the crystal structure of their lipid matrix is a crucial characteristic to obtain high quality SLN formulations. This feature, the formation of the crystal solid state of the lipids of the matrix, depends on the selection and relative proportion of the components, as well as on the preparation method.

Since the composition of the nanoparticle has a great influence in the quality and characteristics of SLNs, the appropriate composition should be chosen to each particular case [6].

To do so, components of the lipid matrix must be carefully selected taking also into account the nature of the drug to be incorporated since it has to be solubilized in the lipid matrix in order to have good entrapment efficiency [11].

The most frequently used materials to form the lipid core are mono-, di-, and triglycerides, fatty acids, fatty alcohols, and waxes, mainly because these lipids present good biocompatibility and their melting point is above body temperature. The formation of a solid core is essential since determines relevant characteristics of SLNs such as controlled drug release or particle stability [7]. Nonetheless, as described by the Thomson–Gibbs equation, the reduction of the diameter of a particle produces a reduction of the melting point of the lipid. Therefore, lipids in nanoparticles do not always behave as bulk lipids and during the production process of lipid nanoparticles supercooled melts can appear (i.e., lipid structures that do not crystallize although being below its melting point) [12]. Consequently, the crystal structure of SLNs cannot be assumed when nanoparticle production process is finished even selecting lipids that are solid at body temperature. Thus, analysis of the solid state of the lipids with infrared spectroscopy or differential scanning calorimetry should be carried out after SLN production in order to determine this characteristic [13]. Supercooled structures will tend to crystallize within short period of time, which may lead to uncontrolled coalescence of lipid droplets and formation of large crystals, leading to uncontrolled particle aggregation and enlargement.

On the contrary, in some special formulations, the melted lipids are stabilized and a liquid crystal structure can be obtained [14,15,16]. These liquid crystals may flow like a liquid, but their molecules may be oriented in a crystal-like manner. Besides, depending on the excipients and the applied method, they can acquire different structures such as cubosomes, hexosomes, spongosomes, micellosomes, and liposomes [17]. These lipid-based liquid crystals can also be applied as biocompatible and efficient drug delivery systems, but they cannot be considered Solid Lipid Nanoparticles (as they do not present a solid state) so they are not in the scope of this review.

**Figure 2:**
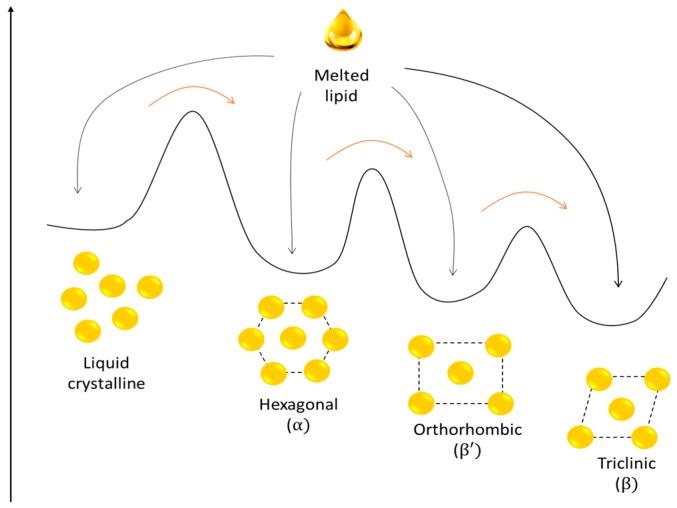
Schematic representation of the energy landscape of different lipid structures and possible polymorphic transformations. Black arrows represent crystallization process after nanoparticle formation; different structures can be formed in the same process. Red arrows represent spontaneous crystal structure transformation during nanoparticle storage.

Another SLN feature related to lipid solidification after nanoparticle production is that depending on the nature of the lipid matrix and the production method, some nanoparticles can crystalize in more than one crystal species [11]. Therefore, in a recrystallization process different polymorphic forms can be generated driving to different internal structures. These polymorphic forms are not long term stable and, after a period of time, lipid crystal structures can be transformed producing more stable structures (Figure 2).

These possible postproduction polymorphic transitions are one of the main concerns about SLN storage stability. Indeed, the polymorphic transitions add structural heterogeneity that impedes the production and characterization of defined DDS, which is needed to fulfill pharmaceutical standards. Additionally, crystal structure modifications can reduce the space to accommodate drug molecules, thus, leading to drug expulsion and lowering drug entrapment efficiency. Furthermore, these transformations can alter main nanoparticle characteristics such as size or shape [7].

**Figure 3:**
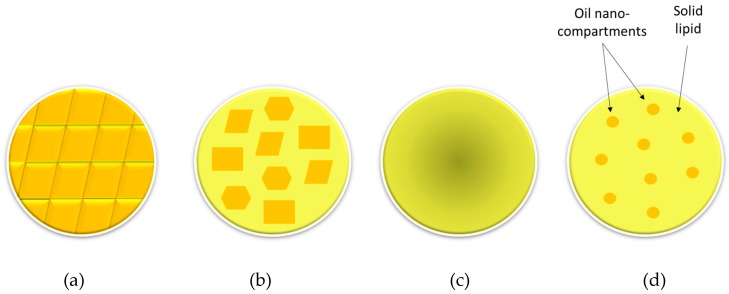
Schematic representation of SLNs and different nanostructured lipid carrier (NLC) types depending on their nanostructures. (**a**) SLN, (**b**) imperfect NLC, (**c**) structureless NLC, and (**d**) multiple oil in solid fat in water O/F/W NLC. Adapted from [18].

Therefore, in some cases, crystallization process of the lipid matrix can be intentionally obstructed in order to avoid drug expulsion related to polymorphic transitions. This can be achieved by adding chemically very different liquid lipids or oils to the solid lipids. Hence, solid lipids cannot form perfect crystals and they solidify producing different nanostructures. Nanoparticles formed by mixing solid lipids and liquid lipids or oils are called nanostructured lipid carriers (NLCs) and are considered as the second generation solid lipid nanoparticles because apart from avoiding drug expulsion they can also improve initial loading capacity of the nanocarrier [18]. Depending on the nature of solid and liquid lipid mixture, different types of nanostructures can be obtained: (i) the imperfect type; (ii) the structureless type; and (iii) the multiple oil in solid fat in water (O/F/W) type (Figure 3) [18].

Paying attention to other components of SLNs, the selection of proper surfactant molecules can also be highly relevant because they affect particle stability and have potential implications in the interactions of designed nanocarriers with cells. Surfactants have amphiphilic structure and are oriented with their polar heads predominantly toward the aqueous phase while keeping their hydrophobic groups away from water. The selection of surfactant molecules mainly depends on the chosen lipid, since they need to be physicochemically compatible [19]. Different amphiphilic molecules can be used as surfactants: monoacylglycerides of long-chain fatty acids, phospholipids, some esters, poloxamers, and polisorbates. Sometimes, additional molecules called cosurfactants are added in order to improve particle stability. Most used cosurfactants are bile salts such as taurodeoxycholate, or alcohols such as butanol or ethanol [6].

After choosing the right composition of SLNs for a determined purpose, interactions between nanoparticle components and their structural organization need to be evaluated. To do so, different biophysical techniques may be adapted to the study of SLNs. In fact, many authors have applied techniques such as infrared spectroscopy, electron paramagnetic resonance (EPR), cryo- and freeze-fracture transmission electron microscopy (Cryo-TEM), small-angle X-ray scattering (SAXS), small-angle x-ray and neutron scattering (SANS), and x-ray powder pattern simulation analysis (XPPSA) either alone or combined [20,21,22,23]. Depending on selected excipients and applied characterization method two types of morphologies have been observed in these works: a platelet-like shape (associated with the stable β-polymorphic state) and a spheroidal or disk-like shape (relate to the α-polymorphic state). The surfactant molecules seem to form close-packed single molecular layers at the interface of the nanoparticles and the dispersion medium [20].

### 1.4. Advantages and Disadvantages of SLNs

In general, one of the main concerns about the use nanoparticles composed of nonbiological compounds (such as inorganic or polymeric nanoparticles) are the potential harmful effects that may cause in our organism, as well as their instability after being administrated [3].

Due to their biocompatible nature, lipid nanoparticles are particularly interesting. In this regard, liposomes represent one of the most used lipid DDS despite their low physical and chemical stability [24].

An alternative to them can be offered by SLNs, formed by biocompatible and biodegradable lipids that are solid at body temperature, making SLNs promising robust nanocarriers for controlled drug delivery [25].

Apart from the abovementioned low toxicity and high stability, SLNs present other interesting advantages, for instance, their high biocompatibility and biodegradability, and the remarkable capacity to incorporate both hydrophilic and lipophilic compounds. In addition, they enable a controlled release of the incorporated drug and they provide chemical protection and, therefore, high stability to the incorporated compound [7]. Liquid crystal nanoparticles (LCNPs) also share these advantageous characteristics and are also considered as very promising nanocarriers [17,26]. However, LCNPs have not been widely applied yet, because of the high costs associated with the massive energy input required in the manufacturing process [16]. 

In this regard, SLNs allow for simple and economical large-scale production [10,27], being some of the most applied preparation methods microemulsion and high-pressure homogenization [6]. 

Another advantage of SLNs is the wide diversity of routes through which their administration is effective: oral, parenteral, rectal, nasal, ocular, etc. [28]. Besides, they can be applied to treat a wide range of diseases, including the use of SLNs in cancer treatment, in gene therapy by using cationic SLNs, or even in the cosmetic field [6,29].

Nevertheless, as mentioned before, these nanoparticles also present some disadvantages related to the recrystallization process, such as a low drug loading capacity and the possibility of drug expulsion during SLN storage. All this, together with the high polydispersity shown by some SLN preparations, may be limiting factors for the industrial production of this type of nanoparticles [28].

### 1.5. Cancer and Cancer Therapy

Cancer is a group of diseases that include the uncontrolled division of cells and resistance to cell death, as well as the ability of these cells to invade other tissues [2]. It represents one of the leading causes of death worldwide [30].

The most extended cancer treatment is the application of chemotherapy through conventional drug administration, but it entails multiple problems, including low drug solubility, low specificity, high toxicity, and low therapy index [2,31]. Another obstacle related to chemotherapy is cancer cells’ resistance to drug treatments. This is known as multidrug resistance (MDR) and it refers to the acquisition of resistance towards a broad spectrum of drugs [32]. Moreover, anticancer drugs administration implies big discomfort to patients since they are essentially administered by injections or intravenously and not by the oral route [33]. Nevertheless, despite all these limitations, nowadays chemotherapy remains as the main cancer treatment [30].

DDS, specifically SLNs, can improve drug effect while overcoming resistance mechanisms. The nanometric size of these systems, together with the possibility of ad hoc modifications, makes them suitable to get through several biological barriers and to deliver drugs at the sites of interest with minimal toxicity [6].

Aside from the already mentioned advantages, the use of SLNs in antitumor treatments could also allow oral administration of drugs and improve the exposure time of cancer cells to medicines in comparison to the most frequent administration methods [33]. This would imply the use of simpler and more convenient therapies for patients.

Considering the impact of cancer worldwide and the need of more efficient therapies, SLNs are presented as particularly promising drug delivery systems for the improvement of cancer chemotherapeutic treatments. Hence, the aims of this review are to gather relevant and current information on the application of SLNs as drug delivery systems in antitumor treatments, to determine the main barriers in cancer treatments and the importance of the mechanisms used by SLNs to improve drug delivery, and to discuss the advances on the application of SLN and remaining challenges in this field.

## 2. Obstacles for an Efficient Chemotherapy

### 2.1. Physiological Barriers 

One of the first considerations to be made in chemotherapeutic treatments are the biological barriers that drugs must overcome to reach their action sites. Focusing on oral administration, despite being considered one of the most proper routes for the administration of medicines, it is not currently an option used in chemotherapy due to the low bioavailability of drugs through this route [34].

After oral administration, the absorption process of molecules must be achieved. The bioavailability of a compound is related to its absorption, which will depend on physicochemical properties of the drug, emphasizing its solubility and permeability, and on physiological factors such as pH conditions, patient’s dietary status, or regional differences in permeability [35].

For absorption to occur it is necessary that the drug gets dissolved in the gastrointestinal medium and then the absorption process itself will take place in the small intestine. After absorption in blood capillaries, the compounds are transferred through the liver before passing into systemic blood circulation, there being exposed to metabolic enzymes. This hepatic metabolism represents the main drawback in the absorption of drugs [29,35]. SLNs and other lipid formulations have been reported to protect compounds against presystemic metabolism and to significantly improve intestinal permeability and absorption, because of their lipophilic nature. In this way, SLNs could overcome this barrier and improve drug administration, especially lipophilic drug administration [36,37].

Nevertheless, there may be other physiological obstacles in the administration of drugs, for instance, when the target of the treatment in therapy against melanomas is the skin. In this case, the stratum corneum acts as an important barrier, blocking penetration of many drugs. However, lipid-based nanoparticles, in general, and SLNs, in particular, are able to enhance drug penetration through the skin, especially those of smaller sizes [38]. There is more evidence that support the use of SLNs for drug delivery through skin layers [39], thus it could be promising to apply this methodology to create nanogel formulations for the treatment of cancer types such as the already mentioned melanomas.

Of special concern in the administration of drugs to the central nervous system to treat brain tumors is the blood–brain barrier (BBB) that represents a tremendous challenge. The BBB is essential to maintain the microenvironment necessary for a suitable neuronal function. To this aim, the BBB is formed by the microvascular endothelium of the brain whose functions, together with some cells such as astrocytes, generate a barrier that separates circulating blood from brain nervous tissue [40].

The improvement of drug delivery to the brain through SLNs is based in the ability of these nanoparticles to stabilize molecules and to increase their bioavailability and permeability. In treatments targeting the central nervous system, the stability of a compound in plasma is essential to maintain an acceptable concentration in order to achieve the desired pharmacological effect. SLNs improve drug bioavailability, allowing the dose that reaches the brain to be higher in comparison to the administration of the free drug. SLNs can also act as vehicles that interact with the barrier, therefore favoring the penetration of drugs that initially could not cross it [41].

Although SLNs enhance drug delivery through the BBB, other strategies based on these nanoparticles could bypass this barrier. This could be achieved by drug administration via nasal or intranasal route, exploiting a nose-to-brain drug delivery path. Nasal administration is a simple method, which would not cause as much disturbs on patients as, for instance, the parenteral route. The optimum nose-to-brain transport occurs through the olfactory epithelium, reaching the olfactory bulb and then the central nervous system [42], and there is evidence reporting that SLNs properly carry out this transport [43].

Finally, there are also barriers against the systemic use of SLNs themselves. For instance, the reticuloendothelial system represents a significant limitation for nanocarriers, since it implies their elimination from blood circulation. The mechanism consists of coating the nanoparticles by plasma components such as albumin or immunoglobulin G, leading to their fast elimination by phagocytic cells. Hence, this may seem to indicate that the effectiveness of nanoparticles will be significantly reduced by the action of phagocytic cells. However, SLNs in the range of 120 to 200 nm and coated by a hydrophilic surface are not rapidly phagocytize by reticuloendothelial system and therefore they can perform their function more accurately [41].

### 2.2. Multidrug Resistance (MDR) 

Together with the heterogeneity of tumors, one of the main challenges limiting the effectiveness of antitumor chemotherapy is multidrug resistance (MDR), which can be intrinsic or acquired by exposure to some compounds [44]. This capacity includes resistance to a numerous compounds with different action mechanisms and chemical structure. MDR mechanisms can be classified into cellular and noncellular, related to biochemical alterations in cancer cells and with tumor microenvironment, respectively [45]. 

**Figure 4:**
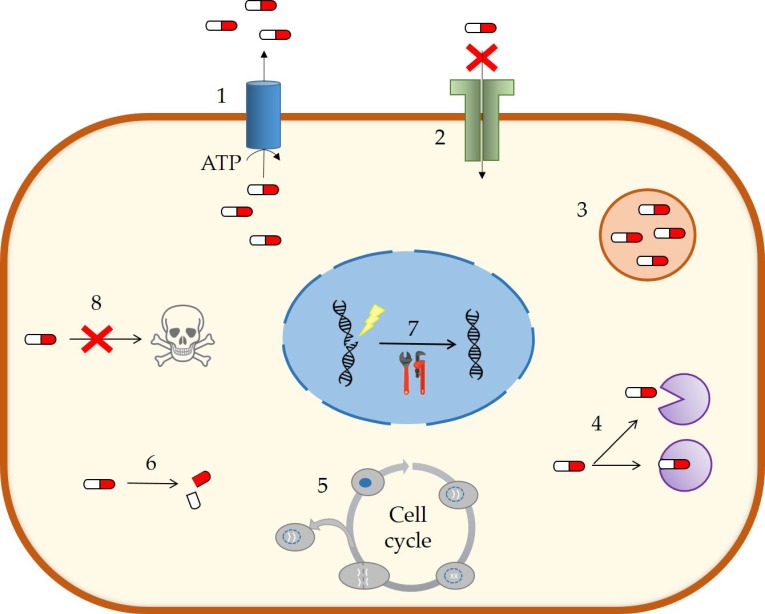
MDR mechanisms in cancer cells. Multidrug resistance can be associated to different biochemical processes: (1) active efflux of compounds, (2) loss of surface receptors or alterations in the cell membrane, (3) drug compartmentalization, (4) alteration of drug targets, (5) changes in the cell cycle, (6) elevated drug metabolism, (7) activation of DNA damage repair systems, and (8) inhibition of apoptosis. Adapted from [46].

Cellular MDR are divided into several mechanisms (Figure 4). Nevertheless, the best characterized and with the greatest contribution to MDR development is the efflux caused by the overexpression of some membrane transporters as a consequence of exposure to cytotoxic drugs [46,47].

The alteration of the efflux transport in MDR is usually a consequence of a high expression of ATP-binding cassette (ABC) transporters. This type of transporters reduces the intracellular concentration of drugs and metabolites using energy obtained from ATP hydrolysis, and its main function is the protection of cells against xenobiotics and toxic compounds [47]. Specifically, P-glycoprotein or ABCB1 has an especially important role as an exporter in cancer cells due to its location and its wide spectrum of substrates. In fact, ~90% of the drugs used in oncology are substrates of this transporter [46,48]. 

On the other hand, focusing on noncellular MDR systems, the pathological conditions of cancer shape the characteristic microenvironment of the tumor. Tumors are structures with a hypoxic nucleus surrounded by proliferating cells. Their microenvironment aids in the proliferation, differentiation, and growth of tumor cells, and it contributes notably to the development of MDR, reducing the action or access of compounds [44].

Many systems based on nanomedicine are able to take advantage of the microenvironment of the tumor for a more specific delivery [2]. In particular, SLNs are capable of overcoming many of these resistance mechanisms because of two relevant features: (i) SLNs allow the cotransport of drugs with molecules that inhibit MDR mechanisms [44] and (ii) SLNs can specially avoid the efflux by exporters such as P-glycoprotein, probably due to a modulation of drug incorporation routes [48]. Thus, approaches based on advantageous characteristics of SLNs for a selective and/or more efficient drug delivery could be crucial for future anticancer treatments using drug delivery systems.

## 3. Drug Delivery of SLNs

A selective delivery of drugs to their sites of action allows an improvement in their effectiveness, as well as the reduction of their side effects [32]. In this regard, it has been described that SLNs can improve their delivery by different mechanisms.

### 3.1. Passive Delivery Mechanisms

**Figure 5:**
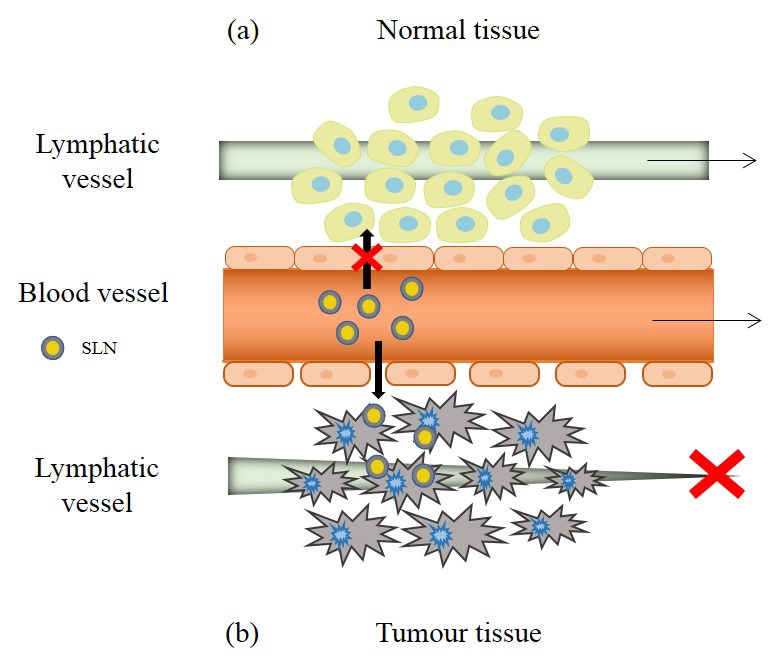
Enhanced permeability and retention effect in tumor tissues. Under normal conditions (**a**) extravasation of the nanoparticles does not occur, but in the tumor region (**b**), the discontinuity of the vascular epithelium and the poor functionality of the lymphatic drainage allow the increase of permeability and retention of SLNs in the microenvironment of the tumor. Adapted from [31].

Specific accumulation of drugs in tumors could be achieved by in situ antitumor injections in the specific areas where the tumor develops. However, in addition to being a more complicated treatment, it is not always possible due to the difficulty of identifying cancerous masses [32]. To overcome this difficulty, some delivery systems, in particular SLNs, allow the targeting of tumor tissues by taking advantage of the characteristics of the tumor microenvironment, due to the so-called enhanced permeability and retention effect (EPR) (Figure 5) [2]. This effect is mainly based in the fast angiogenesis carried out by solid tumors to maintain enough supplies of oxygen and nutrients [31]. 

The angiogenesis process in tumor regions promotes the development of irregular blood vessels with discontinuous epithelium. These discontinuities between epithelial cells, with a size of 100 to 800 nm, allow nanoparticles of certain sizes to move through the interstitial space, being that the base of the increased permeability. In addition, tumor tissues are characterized by a dysfunctional lymphatic system, which implies insufficient lymphatic drainage, leading to the accumulation of nanoparticles in the tumor tissue. This is the basis of increased retention [31,49].

Besides, molecule distribution according to the EPR effect is variable and is determined by three interrelated processes: the extravasation of nanoparticles from the blood vessels, the diffusion of nanoparticles through the tumor tissue, and the interaction with intra- or extracellular targets in the microenvironment of the tumor [49].

Although the EPR effect is presented as a beneficial mechanism to improve the selective delivery of drugs, in recent years it has been shown that the clinical results have not reached the expected potential. This effect presents a high heterogeneity and depends on many factors, such as the type and size of the tumor. Tumors where the greatest accumulation of nanomedicines has been found in relation to the EPR effect are the pancreatic, colon, breast, and stomach tumors [50]. Therefore, when exploiting this phenomenon for a specific delivery with SLNs, it could be necessary to take into account the specific characteristics of the tumor.

### 3.2. Active Delivery Mechanisms

Active delivery mechanisms are focused on the recognition of target molecules such as receptors or transporters overexpressed on the surface of tumor cells. Since the rate of cell proliferation in tumor tissues is high, the demand of nutrients is elevated. Taking into account that most of these nutrients require selective surface transporters to enter the cells, the expression of transporters in tumor cells is consequently increased. Hence, transporter abundance could offer some additional advantages comparing to surface receptors as targets, such as a broader spectrum of substrates [51].

Therefore, the idea of these therapies consists of directing the supply of pharmacological agents selectively to cancer cells by modifying the surface of nanoparticles, thereby minimizing damage to normal cells and avoiding undesired effects [31]. Specifically, the surface modification of nanoparticles allows them to present multiple copies of the ligand of interest, so the binding avidity to the transporter will be high [49].

It is remarkable that despite allowing a greater drug administration and selectivity, it is important that the surface modification does not radically change nanoparticles biodistribution profile. Furthermore, for the ligand–target interaction it is necessary for the nanocarriers to be close to their target and besides, systems with high circulation times are required [49].

There are different ligands that can be used to carry out active drug delivery mediated by SLNs. One of them is hyaluronic acid, since in several types of tumors there can be appreciated an overexpression of its receptors (CD44 and CD168). Thus, it has been confirmed that SLNs loaded with paclitaxel and with their surface functionalized with hyaluronic and pluronic acid (an inhibitor of the efflux transporter P-glycoprotein) show the ability to overcome drug resistance and reduce cell viability in HeLa cervix and MCF-7 breast tumor lines. Moreover, these SLNs increase drug concentration and efficacy in a very significant way in tumor tissues in mice, in relation to SLNs without hyaluronic acid and to the free drug [52].

As a different approach, it has been tested the modification of SLNs loaded with the drug docetaxel with more than one ligand to allow a directed delivery, synergistically combining the previously mentioned hyaluronic acid and tetraiodothyroacetic acid. The latter compound is an analogue of thyroid hormones that has a high affinity for the integrin α_v_β_3_, a molecule overexpressed in tumor endothelial cells. In this way, the modification with tetraiodothyroacetic acid would allow the transport of drugs to the surface of the tumors, enhancing the active delivery capacity of the hyaluronic acid. Moreover, this hypothetical betterment was studied in vitro, showing high incorporation and reduced viability in B16F10 mouse melanoma cells (expressing α_v_β_3_ and CD44). These SLNs were also tested in vivo, in mice with lung tumors originated in situ and with implanted melanoma tumors, observing a remarkable inhibition of tumor growth [53].

Another example could be the addition of sugars such as galactose to the nanoparticles surface for a more efficient cell uptake of anticancer agents due to lectin receptors. In this way, galactosylation of SLNs has been verified as an approach that favors their incorporation and cytotoxicity, for example, when used for the transport of doxorubicin against A549 human lung epithelial cancer cells. Besides, these SLNs have also been shown to improve drug distribution in tumors in mice, induced using the A549 cell line [54].

### 3.3. Codelivery Mechanisms

A possibility to overcome drug resistance in tumor cells is the addition of two different compounds to nanoparticles: the antitumor drug and an agent responsible to act against MDR mechanisms. Some of the main strategies include, for instance, small interfering RNA (siRNA) to silence gene expression of ABC transporters, microRNAs (miRNAs) that allow post-transcriptional regulation of genes or inhibitory compounds of these exporters [44,55].

For example, among those mentioned inhibitory compounds, pluronic polymers have been shown to be effective in reducing resistance mechanisms, especially in the inhibition of efflux transporters overexpressed in the membrane of cancer cells. In addition to compounds capable of reversing MDR mechanisms, it could also be adequate the application of compounds that prevent their appearance or delay it. It has been studied the efficiency of different preventive agents coadministered with cytotoxic drugs, and the combination seems to increase the effectiveness of chemotherapy. Among them, dexrazoxane is capable of suppressing the stimulation of P-glycoprotein expression that drugs can induce, thus preventing the development of MDR [56].

From another perspective, cotransport also includes the combined delivery of different types of drugs. This could imply an optimization in the treatments, being able to be administered at the same time and allowing a synergic effect in the action against tumor cells. The effectiveness of a combined SLN drug delivery of the anticancer drug paclitaxel and the heat shock protein 90 (Hsp90) inhibitor called tanespimycin has been confirmed. It has been shown that the inhibition of Hsp90 can repress the expression of receptors that favor the growth of gastric tumors. The study concluded that this codelivery results in an enhanced antitumor effect, reducing cell viability in different human gastric cell lines and the weight and size of gastric tumors in mice [57].

As an interesting approach, it is possible to combine active targeting by SLN functionalization and codelivery using different drugs. This was carried out using SLN modified with polyethyleneglycol-distearoyl-phosphatidylethanolamine and functionalizing them with the trans-activating transcriptional activator or TAT, a peptide that could allow a better penetration of the nanocarriers into cells. The drugs for the codelivery were paclitaxel and cisplatin, being the last one conjugated to α-tocopherol succinate, a derivative of vitamin E. Functionalization and codelivery showed a synergic effect by improving cellular uptake in vitro in HeLa cells and antitumor activity in vivo, by reducing the volume of cervical tumors in mice [58].

## 4. Drugs for Antitumor Treatments

**Figure 6:**
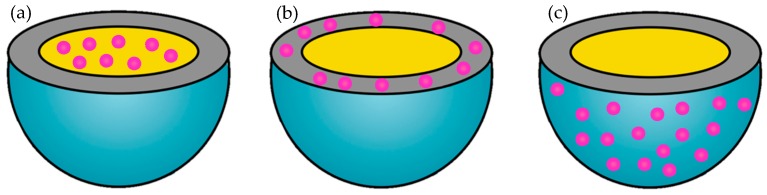
Drug distribution in solid lipid nanoparticles. Possible ways of incorporation of a drug (pink) in a SLN: (**a**) homogeneous dispersion in the lipid matrix, (**b**) incorporation in the shell of the matrix, or (**c**) distribution in the outer shell.

SLNs allow the incorporation of multiple drugs—both hydrophilic and lipophilic. Depending on the nanoparticles composition and the method of preparation, the drug can be incorporated in three ways (Figure 6): (i) it can be dispersed homogeneously in the lipid matrix, (ii) it can be incorporated into the shell surrounding said matrix, or (iii) it can be distributed in the outer shell [2].

In antitumor chemotherapy, the drugs used can be divided into alkylating agents, antimetabolites, natural products, or hormonal compounds [30]. In this section, it will be exemplified that SLNs can incorporate all of these types.

Among the alkylating agents temozolomide can be outlined. This compound has three nitrogen atoms adjacent to a heterocyclic ring that give an important anticancer activity. Temozolomide has proved to be more effective when administered by SLNs in the treatment of melanomas, providing a notably greater cytotoxicity in JR8, A2058 human, and B16-F10 mouse melanoma cell lines, compared to free temozolomide. Moreover, in vivo application of temozolomide-loaded SLNs in mice with melanoma tumors verified that the use of these nanocarriers reduced tumor size by 50%, while the drug in solution showed nonsignificant effects [59].

The prodrug capecitabine is an antimetabolite that can be transformed into fluorouracil and it is used against different types of cancers (breast or colon cancer), but it has many harmful effects, such as cardiotoxicity or nausea induction. Capecitabine can be effectively loaded into SLNs, and this formulation was evaluated both in vitro and in vivo. Cytotoxicity assays were performed with HT-29 human colon cancer cells, and capecitabine incorporated into SLNs showed greater cytotoxic capacity that the free drug. Moreover, it is worth mentioning that the SLN delivery system led to enhanced bioavailability of the drug and reduced tumor histopathological alterations in rats with 1,2-dimethylhydrazine induced colon cancer [60].

Docetaxel is a lipophilic natural product with antimitotic activity and low solubility in water, so formulations with SLNs could improve its administration. This was demonstrated in pharmacokinetic studies by measuring its plasma concentration in rats, and it was concluded that SLNs allow a greater absorption and less elimination. In addition, these systems were able to improve the induction of cytotoxicity in MCF-7 breast tumor cells in relation to the drug in its commercial form (in micelles) and in its free form [61].

Doxorubicin is another natural agent of great relevance as an anticancer compound. Its administration shows several associated disadvantages such as heart problems, in addition to the development of resistance by cancer cells. It has been observed that the incorporation of doxorubicin in SLNs is possible and in combination with α-tocopherol succinate (which also presents anticancer activity), it shows high cytotoxicity and high uptake capacity in resistant MCF-7 human breast and NCI ovarian cancer cells [62].

There are more natural products that are used in cancer therapy, such as the aforementioned paclitaxel, which causes cellular apoptosis [30]; another example is curcumin, a natural compound with anti-inflammatory and antioxidant properties that also interferes with transduction routes and induces cell cycle arrest or apoptosis [56,63].

It has also been reported that hormonal compounds can be incorporated into SLNs. For instance, SLNs loaded with the estrogen receptor targeting tamoxifen have been tested against tamoxifen-resistant MCF-7 breast cancer cells. The cytotoxic activity of tamoxifen and tamoxifen-SLN were similar in nonresistant cells, although the reduction of cell viability by tamoxifen-SLN appeared at earlier times. On the other hand, the treatment with free tamoxifen did not show antitumor capacity against drug-resistant cells, whereas SLNs with tamoxifen were much more efficient and aggressive [64]. 

## 5. Effective Use of SLNs in Different Types of Tumors

It has been documented that SLNs are useful in the treatment of a wide variety of tumors. In this section, to illustrate the applications and potential of SLNs as carriers of antitumor drugs, we will focus on several types of tumors with significant prevalence and representing high threats, in order to illustrate the applications and/or potential of SLNs as carriers of antitumor drugs: breast, lung, colon, hepatic, and brain tumors [65]. The examples presented in this section are summarized in Table 1.

### 5.1. Breast Tumor

Breast cancer is the most common type of cancer in women and its prevalence keeps increasing over the years [65]. There are different examples that reflect the effective use of SLNs to treat these tumors. 

For instance, it has been possible to carry out a delivery of a specific miRNA-200c in cationic SLNs to avoid the resistance of breast cancer cells, and this was used to increase response to the drug paclitaxel administered by NLC. The levels of this miRNA are decreased in breast cancer stem cells, and its delivery by SLNs could lead to a reduced expression of class III beta-tubulin, which would imply an increased effectiveness of microtubule-targeting drugs (such as paclitaxel). In this study, MCF-7 breast cancer cells were used to generate mammospheres, and SLNs were able to transfect these cells more efficiently than Lipofectamine or the free miRNA-200c, without affecting cell viability or the morphology of mammospheres. Finally, it was possible to conclude that the use of this therapy improved the drug half maximum inhibitory concentrations (IC_50_) value of paclitaxel-loaded NLC against cancer cells, and that it could be an adequate strategy for miRNA delivery in the treatment of breast tumors [55].

In other works, paclitaxel-loaded SLNs were used against drug-resistant breast cancer cells. Paclitaxel-SLN activity against MCF-7 drug-resistant and drug sensitive cells was compared to the use of other formulations, such as dimethyl sulfoxide solubilization and Cremophor EL vehicles (commercial formulation). The evaluation of concentration-dependent cytotoxicity indicated that SLNs with paclitaxel notably improved the IC_50_ concentration in drug-resistant cells. Besides, these nanocarriers provided an enhanced cellular uptake in relation to the other formulations especially in drug-resistant cells, proving that SLNs are effective in avoiding multidrug resistance mechanisms in breast cancer cells [66]. 

There are other studies that have also corroborated the effectiveness of SLNs, such as their application as curcumin carriers against the breast cancer cell line MDA-MB-231. The results of this work showed a strong increase in the drug uptake capacity of the cells when curcumin was administered into the SLNs. Also, curcumin-SLN promoted a higher decrease in cell viability and an increase in apoptotic cells than curcumin diluted in dimethyl sulfoxide [63].

Additionally, the efficacy of SLNs against breast tumors in vivo has been analyzed as well, further demonstrating the importance of this DDS against breast tumors. For these experiments, SLNs were loaded with methotrexate and functionalized with fucose to achieve an active targeting of tumors. First, methotrexate showed a significantly increased cytotoxic effect in MCF-7 cells when incorporated into fucose-SLNs than when incorporated into SLNs or alone. Also, methotrexate-loaded SLNs, especially those modified with fucose, showed a greater concentration of the drug in tumor tissues together with an improved antitumor effect in rats with induced breast cancer. As results from methotrexate fucose-SLN and SLNs were more favorable than those from free methotrexate, it can be concluded that in this case SLNs improved the treatment against breast tumors [67].

### 5.2. Lung Tumor

Lung tumors are one of the main types of cancer and the first cause of death due to cancer in the United States [65], thus, the achievement of an effective and specific treatment is challenging. For instance, SLNs have been analyzed as delivery systems of the anticancer compound naringenin. It has been observed that although this system did not reduce cell viability in A549 lung epithelial cells (probably due to the use of naringenin, which has not been reported to be effective against A549 cells), naringenin-SLN showed a good cellular uptake pattern. Regarding biodistribution studies in rats, the administration of naringenin with SLNs by intratracheal instillation ameliorated the pharmacokinetic parameters of the drug, including mean residence time or maximum plasma concentration [68].

Lung tumors have special interest because they can be treated using SLNs administered directly into lungs by inhalation. However, this type of therapy is associated to certain limitations, such as short residence times and little tolerance as a consequence of an uncontrolled drug release. In this regard, paclitaxel loaded into SLNs has been used to revert some of these limitations, being those prepared SLNs coated with a polymer formed by folate-poly(ethylene glycol) and chitosan. These studies demonstrated that SLNs reduced the IC_50_ value in vitro against M109HiFR lung cancer cells. It was also determined that SLNs were able to increase the drug concentration in vivo in lungs of healthy and sick mice, when administered by inhalation [69].

Other researchers have reported that the incorporation of the poorly-soluble compound erlotinib into SLNs could be useful against A549 cells and for inhalation administration. In vitro experiments showed that the cytotoxic effect of the free drug was lower than the effect of the drug encapsulated in SLNs. Furthermore, SLNs loaded with erlotinib presented an adequate aerosol dispersion performance, indicating that this system could be suitable for pulmonary delivery [70].

### 5.3. Colon Tumor

Colon cancer has a remarkable incidence in society and it is the third cause of death related to cancer in the United States [65]. Therefore, finding strategies to act against this condition is essential. Some works have demonstrated that SLNs could be a powerful method to treat colon tumors. As an example, SLNs have been used to deliver omega-3 polyunsaturated fatty acids (α-linolenic acid or docosahexaenoic acid) against HT-29 and GCT116 adenocarcinoma cells. In this experiments, the SLNs proved to actively inhibit cell growth in a dose-dependent manner and more drastically than the free fatty acids. More specifically, the incorporation of the fatty acid in SLNs slightly incremented apoptosis activation and it strongly reduced cell proliferation in HT-29 cells [71].

Other studies have developed SLNs functionalized with folic acid for the delivery of oxaliplatin, as folate receptors are overexpressed in colorectal carcinomas. After testing the antigrowth potential of this formulation against HT-29 human colon cancer cells, oxaliplatin incorporated in SLNs with folic acid showed the greatest anticancer capacity, comparing to nonfunctionalized SLNs and the free drug [72]. 

### 5.4. Hepatic Tumor

Hepatic tumors represent also a considerable portion of deaths related to cancer and new therapeutic approaches are needed [65]. Superparamagnetic iron oxide nanoparticles (SPIONs) can be incorporated into SLNs to control delivery using an external magnetic field. Taking this into account, SLNs were loaded with the drug sorafenib and SPIONs were added to HepG2 human hepatocarcinoma cells. This drug delivery strategy showed a significant cytotoxic effect, but still not as strong as the free drug. Despite this, the cellular uptake provided by SLNs and magnetic targeting experiments evidenced that these nanoparticles may ameliorate hepatocarcinoma treatment [73]. 

In another work, SLNs with different lipid compositions were generated and the compound linalool was added to those formulations. The antitumor capacity of these agents was verified not only in a HepG2 human hepatocarcinoma cell line, but also in A549 lung adenocarcinoma cells. Focusing on HepG2 cells, SLNs with linalool presented strong antiproliferative activity, which was dose- and time-dependent [74].

### 5.5. Brain Tumor

Brain cancer represents a strong impact on the life of diagnosed patients and it supposes a serious problem for health systems [40]. There are studies where SLNs have proven their capacity to improve the treatment of this disease. One of those studies focused on the use of SLNs to deliver the drug indirubin against a human U87MG glioblastoma-astrocytoma cell line. SLNs increased the cytotoxic effect of the drug (especially in acidic conditions), demonstrating that these nanoparticles have the potential to be applied against brain cancer cells [75].

As previously mentioned, it is complicated to treat brain tumors because the blood–brain barrier represents a significant obstacle. To enhance drug delivery in these therapies, the surface of SLNs can be modified with molecules that target receptors highly expressed in the blood–brain barrier. For instance, SLNs can be coated with apolipoprotein E (ApoE), a molecule specifically recognized by the receptors of low- or very low-density lipoproteins (LDL or VLDL). These receptors are expressed in cells of the blood–brain barrier, and targeting them with ApoE would allow an active cellular uptake of ApoE-SLN. Thus, this approach could increase the accumulation of nanoparticles in the brain [76,77].

SLNs that combine this ApoE surface modification together with the incorporation of the drug methotrexate (as its lipophilic ester didoceylmethotrexate) have proven a reduction capacity of glioblastoma tumors from F98/Fisher rat models. Besides, they exhibited a reduced elimination of the drug from plasma and brain compared to the administration of the compound in free form [76]. It has also been verified that ApoE-modified SLNs enhanced cellular incorporation and transcytosis through the blood–brain barrier. When SLNs were coated with ApoE using DSPE-PEG-avidin or palmitate-avidin as linkers, they were able to increase cellular uptake in a hCMEC/D3 cell monolayer simulating the characteristics of the BBB [77].

### 5.6. Other Tumors

We have focused on the already-mentioned type of tumors due to their impact and prevalence, but SLNs are efficient against other type of cancers. One of these types is leukemia in which it has been evaluated the introduction of the lignin AP9-cd into SLNs to assess its anticancer activity. SLNs with AP9-cd presented enhanced cytotoxic effects comparing to AP9-cd alone against human leukemia Molt-4 cells. Moreover, AP9-cd incorporated into SLNs showed stronger antitumor capacity in experimental Ehrlich ascites tumor model [78].

Another example would be the action of SLNs against prostate cancer. SLNs have been loaded with retinoic acid, which presents very low aqueous solubility and the anticancer effect of retinoic acid in SLNs was evaluated in LNCaP prostate cancer cells. Although free retinoic acid produced a greater reduction in cell viability, the effect of retinoic acid-SLN was also relevant, and furthermore, the use of SLNs solves the poor solubility of the drug [79]. 

In addition, SLNs based on α-tocopheryl linolenate were obtained in order to generate a novel lipophilic formulation with intrinsic antitumor activity. These SLNs were further loaded with the omega-3 α-linoleic acid in order to compare the synergic effect of α-tocopheryl linolenate-based SLNs and α-linoleic acid. In vitro studies with C32 human melanoma cells indicated that both SLN preparations showed cytotoxic activity in relation to the free compounds (α-tocopherol and α-linoleic acid), proving once more the potential of SLNs [80].

## 6. Critical Discussion and Future Perspectives

In this work, we have reviewed state-of-the-art of SLN characteristics, advantages and disadvantages, as well as the latest works related to application of SLNs to improve the actual efficiency of anticancer drugs in different tumor types.

As detailed throughout this work, SLNs represent an innovative drug transport system that allows for overcoming a great number of difficulties related to drug administration. Nevertheless, it is also remarkable that these nanoparticles themselves have certain disadvantages or difficulties that show the need to optimize some of their characteristics.

One of the main concerns related to SLN production is that polymorphic modification on the crystal structure of SLNs can occur during storage time. This could lead to drug expulsion and subsequent lowering of the drug loading capacity. Besides, polymorphic modification can induce drastic changes in nanoparticle size and shape and these changes could destabilize the SLN suspension, triggering particle aggregation. However, it has been described that SLNs formed by mixed lipids allow the incorporation of larger amounts of drugs. Besides, this heterogeneity of the solid lipid matrix also improves nanoparticles stability during storage, and thus, using mixtures of lipids could avoid both problems [6]. To overcome these difficulties, new strategies have been explored. Specifically, in NLCs formed by mixing solid lipid with liquid lipids, polymorphic modifications can be reduced and drug loading capability can be increased. Thus, NLCs were produced in order to overcome the difficulties related to SLNs. Nonetheless, instability problems such as particle aggregation or unexpected gelation may occur in both SLNs and NLCs.

The abovementioned problems could be reduced in case we completely understood nanoparticle formation process and structure because we could rationally modify the components and the processes to form SLNs in order to obtain better characteristics. Unfortunately, due to reduced size and the complexity of colloidal populations it is highly difficult to measure solidification process, particle shape, and nanostructure or particle dynamics. Some advanced biophysical techniques such as cryo- and freeze-fracture transmission electron microscopy, SAXS, and SANS are being applied to study SLN structures and more research in this field is needed to obtain systematic understanding of the process of SLN formation. Improving the application of these techniques would produce better knowledge and this would provide us the tools to perform a rational design of SLNs to achieve tailored improvement in the efficiency of a given therapeutic treatment. 

Moving onto tumor therapies, the capacity of SLNs to overcome a great part of MDR mechanisms has been reported [48]. However, this may also indicate that these nanoparticles will not be effective in any type of tumor. Instead, it will be dependent on the resistance characteristics developed by tumor cells, since there will be resistance mechanisms that SLNs will not be able to revert. 

Despite this, there are numerous studies that describe promising results when encapsulating different drugs in SLNs and applying them as a strategy against cancer cells resistant to the drug. These studies support the idea that these nanocarriers can effectively reverse the main resistance mechanisms [47,48,64]. Nevertheless, it could be adequate to evaluate resistance factors of each tumor to anticipate SLN efficacy in each specific treatment. 

Another aspect that seems to generate controversy in the application of SLNs is the variability of the EPR effect [50]. This effect depends on the characteristics of each tumor, thus, the formation of a general strategy for cancer chemotherapies is not possible and therapies with active delivery mechanisms remain more attractive. In this way, the use of active delivery systems could also be reinforced by the ability of SLNs to improve their delivery in tumor regions where the microenvironment of the tumor is favorable for that to happen [2].

All in all, there are many drugs that have been incorporated in SLNs with positive results [30,59,60,61,62,63,64], as well as tumors in which their action has been verified [58,63,66,67,68,69,70,71,72,73,74,75,76,77,78,79,80]. These are the main supports for the advance in research on the use of SLNs in tumor treatments.

Overall, SLNs seem to be a promising strategy in the fight against cancer diseases. Therefore, as a future perspective, a line of research focused on the development of surface-modified SLNs could have great interest for an active and specific delivery in different types of tumors, with different resistance mechanisms associated. This could be very useful in order to find individualized and highly effective therapies.

## 7. Conclusions

SLNs provide a biocompatible DDS that can be used to incorporate a wide variety of drugs and to treat different types of tumors while overcoming resistance mechanisms in cancer cells. In addition, SLNs facilitate the cellular uptake of the incorporated drugs by the modulation of passive, active, and cotransport mechanisms and are able to overcome biological barriers. Nevertheless, as we have mentioned in this work, better understanding of SLN formation, nanostructure, and drug loading would lead us to the rational design of tailored SLNs for each cancer treatment.

## Figures and Tables

**Table 1:** Use of SLNs against different types of tumors. Summary of studies related to the use of SLNs to improve the action of anticancer drugs or agents. Studies applying SLNs against breast, lung, colon, and brain tumors are included.

Tumor Type	SLN Composition	Drug Incorporated	Reference
Breast	DOTAP, monestearin and Poloxamer 188	miRN-200c, combined with paclitaxel-NLC	[58]
	Trimyristin, egg L-α-PC DSPE-methylPEG-2000	Paclitaxel	[66]
	Cholesterol and Poloxamer 188	Curcumin	[63]
	Gelucire, stearyl amine, phospholipid 90 NG, Tween 80 and fucose coating	Methotrexate	[67]
Lung	Glycerol monostearate, egg-PC, Poloxamer 188, Tween 80	Naringenin	[68]
	Glycetyl stearate, cholesterol, D-α-tocopherol PEG 1000 succinate, sodium taurocholate, and F-PEG-HTCC	Paclitaxel	[69]
	Glycerol monostearate, Poloxamer 188, and transcutol	Erlotinib	[70]
Colon	Resveratrol, stearic acid, sodium taurocholate, Tween 80 and butanol	Omega-3 PUFA	[71]
	Tristearin, Lipoid S75, Tween 80, DSPE, and folic acid	Oxaliplatin	[72]
Liver	Cetyl palmitate and Tween 80	Sorafenib tosylate and SPIONs	[73]
	Myristyl myristate/cetyl esters/cetyl palmitate, and Pluronic F68	Linalool	[74]
Brain	Cetyl palmitate and polysorbate 80	Indirubin	[75]
	Behenic acid and PVA 9000	Methotrexate	[76]
	Cetyl palmitate, Tween 80, ApoE, DSPE-PEG-avidin, and/or palmitate-avidin	-	[77]
Leukemia	Soy lecithin, Tween 80 and Compritol 888 ATO	AP9-cd	[78]
Prostate	Stearic acid, and Poloxamer 188	Retinoic acid	[79]
Melanoma	α-Tocopheryl linolenate, sodium taurocholate, Tween 20, and butanol	α-Linolenic acid	[80]

Abbreviations: DOTAP: 1,2-dioleoyl-3-trimethylammonium-propane; PC: phosphatidylcholine; DSPE: 1,2-distearoyl-sn-glycero-3-phosphoethanolamine; PEG: polyethilene(glicol); F-PEG-HTCC: folate-poly(ethylene glycol)-N-[(2-hydroxy-3-trimethyl-ammonium) propyl] chitosan; PUFA: polyunsaturated fatty acids; PVA: hydrolyzed polyvinyl alcohol 9000–10,000 Mw; SPIONs: superparamagnetic iron oxide nanoparticles.
